# Posterior circumferential decompression for multilevel thoracic ossification of the posterior longitudinal ligament: a case report and literature review

**DOI:** 10.3389/fsurg.2026.1912076

**Published:** 2026-10-06

**Authors:** Lei Ren, Hongju Liu, Chuiguo Sun, Jieyu Liang, Chao Xu, Weiye Ti

**Affiliations:** 1 Joint Sports Medicine Department, The Second Affiliated Hospital of Xinjiang Medical University, Urumqi, China; 2 Neurosurgery Department, China-Japan Friendship Hospital, Beijing, China; 3 Orthopedic Department, Peking University Third Hospital, Beijing, China; 4 Department of Hand, Foot and Vascular Surgery, Peking University Shenzhen Hospital, Shenzhen, China; 5 Department of Bone and Joint, Liwan District Orthopedic and Traumatology Hospital, Guangzhou, China

**Keywords:** cerebrospinal fluid leak, multilevel decompression, spinal cord de-tension, spinal shortening, T-OPLL

## Abstract

Multilevel thoracic ossification of the posterior longitudinal ligament (T-OPLL), especially when it involves more than five consecutive vertebrae, remains surgically challenging. In recent years, a strategy focused on “spinal cord de-tension” has been proposed for such extensive involvements. This report describes a 52-year-old woman who presented with progressive thoracic myelopathy due to continuous OPLL from T3 to T10, causing severe canal stenosis. The surgical approach included a posterior laminectomy from T4 to T10, circumferential decompression at the most compressed level (T8–9), and long-segment instrumentation from T3 to T11. A postoperative cerebrospinal fluid leak was suspected but managed conservatively with controlled drainage and bed rest. By the 12-month follow-up, the patient's neurological status had improved markedly: her modified Japanese Orthopaedic Association (mJOA) score rose from 5 to 10, corresponding to a Hirabayashi recovery rate of 83.3%. Radiographs showed adequate decompression, solid fusion, and no implant failure. Taken together, for symptomatic, ultra-long segment T-OPLL, posterior circumferential decompression combined with long-segment fusion under the spinal cord de-tension principle appears to be highly effective. Spinal shortening achieved through parallel osteotomy and compression provides indirect longitudinal de-tension, complementing direct ventral decompression while maintaining stability. Careful surgical technique and systematic management of complications like cerebrospinal fluid leakage remain essential for a good outcome. This approach offers a favorable risk-benefit balance in these complex cases.

## Introduction

Thoracic ossification of the posterior longitudinal ligament (T-OPLL) is a significant cause of severe thoracic myelopathy, especially when involving long segments (typically ≥3 vertebrae), posing considerable challenges in surgical decision-making and execution (1, 2). The optimal surgical strategy for such complex cases remains debated, with anterior, posterior, and combined approaches each presenting distinct trade-offs (3, 4). To optimize the surgical outcomes and minimize complication risks of multilevel T-OPLL, the concept of “spinal cord de-tension” has emerged as a crucial surgical strategy. This integrated approach encompasses wide posterior laminectomy, selective circumferential decompression, and targeted kyphosis reduction or spinal shortening to decrease the longitudinal tension on the spinal cord. We report a case of ultra-long segment T-OPLL involving seven consecutive vertebrae, successfully managed with the spinal cord de-tension strategy through wide posterior laminectomy, targeted circumferential decompression, and spinal shortening. The patient developed a probable postoperative cerebrospinal fluid (CSF) leak that was successfully treated conservatively, leading to good neurological recovery at the 12-month follow-up. Combined with a literature review, this study highlights the technical nuances, complication management, and clinical value of this surgical approach for similar cases.

## Case presentation

The patient was a 52-year-old female with a body mass index (BMI) of 23.88 kg/m^2^ who developed intermittent distending pain in the chest and back, along with sensory abnormalities in the upper abdomen and perineal region, three months after a mountain climbing episode. Her symptoms were inadequately relieved by self-administered oral NSAIDs and bed rest. She denied any bowel/bladder dysfunction, local trauma, prior spinal surgery, history of similar symptoms, radiating pain, radicular symptoms, or lower limb numbness/weakness. Her past medical history included chronic gastritis, hypothyroidism, hepatic hydatid cyst resection, and right pulmonary nodule resection. During the initial outpatient evaluation, she exhibited an intermittent claudication gait. Physical examination findings are summarized in Table 1. Preoperative neurological status was graded as ASIA D, with a modified Japanese Orthopaedic Association (mJOA) score of 5 points.

**Table 1:** Summary of physical examination findings.

Examination category	Findings
Spinal inspection	No scoliosis or kyphotic deformity.
Palpation	Tenderness over thoracic spinous processes.
Abdominal reflexes	Normal at T7–T8 and T9–T10; weakened from T11 to T12.
Sensation	Decreased in the right perineal region.
Motor strength (lower limbs)	Right: Grade V; Left: Grade IV.
Tone & sensation (lower limbs)	Normal in both limbs.

Spinal inspection refers to visual assessment of spinal alignment in the coronal and sagittal planes. Palpation refers to evaluation of tenderness over the spinous processes. Abdominal reflexes are tested by stroking the abdominal skin to elicit a superficial abdominal muscle contraction. Sensation is assessed by light touch and pinprick stimuli. Motor strength is graded according to the Medical Research Council (MRC) scale from 0 (no contraction) to 5 (normal strength). Tone is assessed by passive movement of the lower limbs, and sensation in the lower limbs includes both superficial and deep sensation.

Thoracic CT revealed ossification of the posterior longitudinal ligament (OPLL) from T3 to T10, causing secondary spinal canal stenosis and associated degenerative changes (Figure 1). The diagnosis of thoracic OPLL and spinal stenosis was established, leading to hospital admission. Upon admission, she received intravenous and oral analgesics along with neurotrophic medication, and a senior spine surgeon conducted a condition assessment on the same day. Combined with thoracic MRI findings showing OPLL from T3 to T10 with secondary spinal canal stenosis, spinal cord compression at the T8–T9 disc level, and thoracic degenerative changes, supplemental diagnoses included thoracic myelopathy and incomplete paralysis. The attending physician discussed with the patient, considering the current thoracic myelopathy and lower limb sensory and motor dysfunction, and concluded that conservative treatment could not relieve spinal cord compression, recommending surgical intervention. The patient agreed to undergo posterior laminectomy and circumferential decompression.

**Figure 1:**
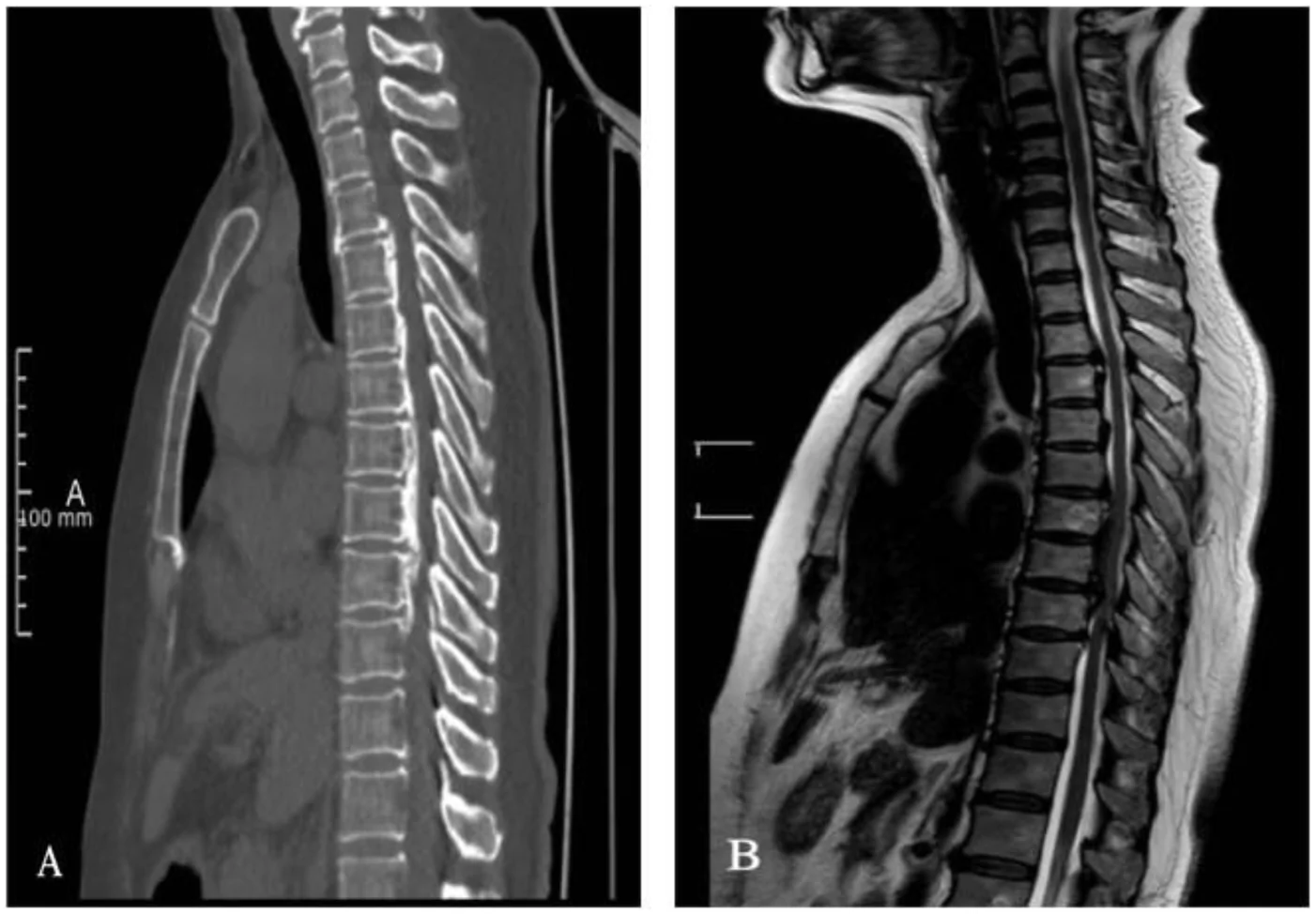
Imaging of the continuous T3–T10 OPLL. Preoperative sagittal CT **(A)** and sagittal MRI **(B)** illustrating the extent of ossification and the concomitant thoracic myelopathy (sagittal thoracic CT scale bar = 400 mm).

The procedure was performed with the patient in the prone position under general anesthesia, utilizing continuous intraoperative neurophysiological monitoring. Image-guided spinal navigation was employed to confirm anatomical landmarks and surgical levels. A midline skin incision was made, and the posterior elements from T3 to T11 were exposed. Pedicle screws were first inserted under fluoroscopic guidance at T3 and T10. A wide laminectomy from T4 to T10 was then performed. The spinous processes were removed with rongeurs, followed by *en bloc* laminectomy using an ultrasonic bone scalpel (Figure 2). After initial posterior decompression, the ligamentum flavum was noted to be thickened and ossified, with attenuated spinal cord pulsation.

**Figure 2:**
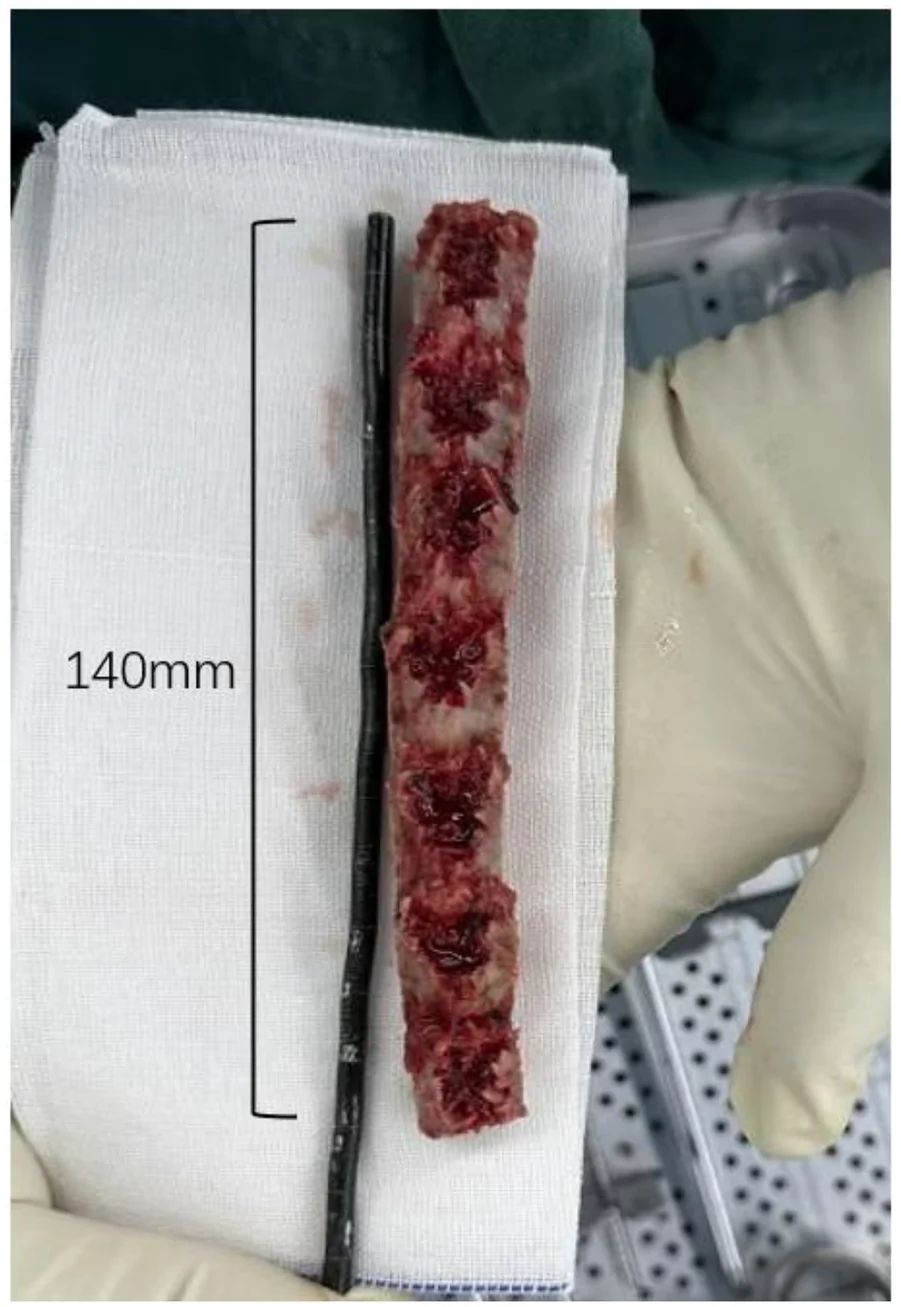
Intraoperatively resected *en bloc* laminae from T4–T10 (total length of the specimen ≈ 140 mm).

Subsequently, focused circumferential decompression was carried out at the most severely compressed level (T8–9). During dissection of the adherent, ossified ligamentum flavum, a small dural tear occurred, which was promptly managed with gelatin sponge packing. The bilateral facet joints and pedicles were undercut using a high-speed drill. The ossified posterior longitudinal ligament was meticulously separated from the dura with a nerve dissector and resected, achieving complete ventral decompression and restoration of satisfactory dural pulsation (Figure 3). Subsequently, a focal parallel osteotomy was performed at the T8–9 intervertebral space. In accordance with the spinal cord de-tension strategy, because the patient's preoperative T4–T12 Cobb angle was less than 25°, indicating normal physiological kyphosis, the angle was maintained. Interbody fusion was performed purely with autologous bone graft without a cage. Pre-contoured rods were then secured to the pedicle screw constructs from T3 to T11. Deliberate longitudinal compression was applied gradually across the T8–9 osteotomy site under continuous multimodal intraoperative neurophysiological monitoring, including motor evoked potentials (MEPs) and somatosensory evoked potentials (SSEPs). The compression was performed incrementally, with the surgical team pausing at each increment to confirm stable monitoring signals before proceeding further. This manoeuvre achieved approximately 2–3 mm of local spinal shortening, as estimated by intraoperative fluoroscopic measurement of the reduction in the intervertebral space. The autologous bone graft, which had been tightly impacted into the osteotomy gap prior to rod placement, was firmly secured under the sustained axial compression. This compression not only reduced the longitudinal cord tension (de-tension) over the anterior ossified mass but also provided immediate mechanical stability to the graft by maintaining close contact with the endplates. Final intraoperative imaging confirmed appropriate implant positioning (Figure 4). A subfascial drain was placed before wound closure. The total estimated blood loss was 700 mL, with 250 mL of autologous blood transfused.

**Figure 3:**
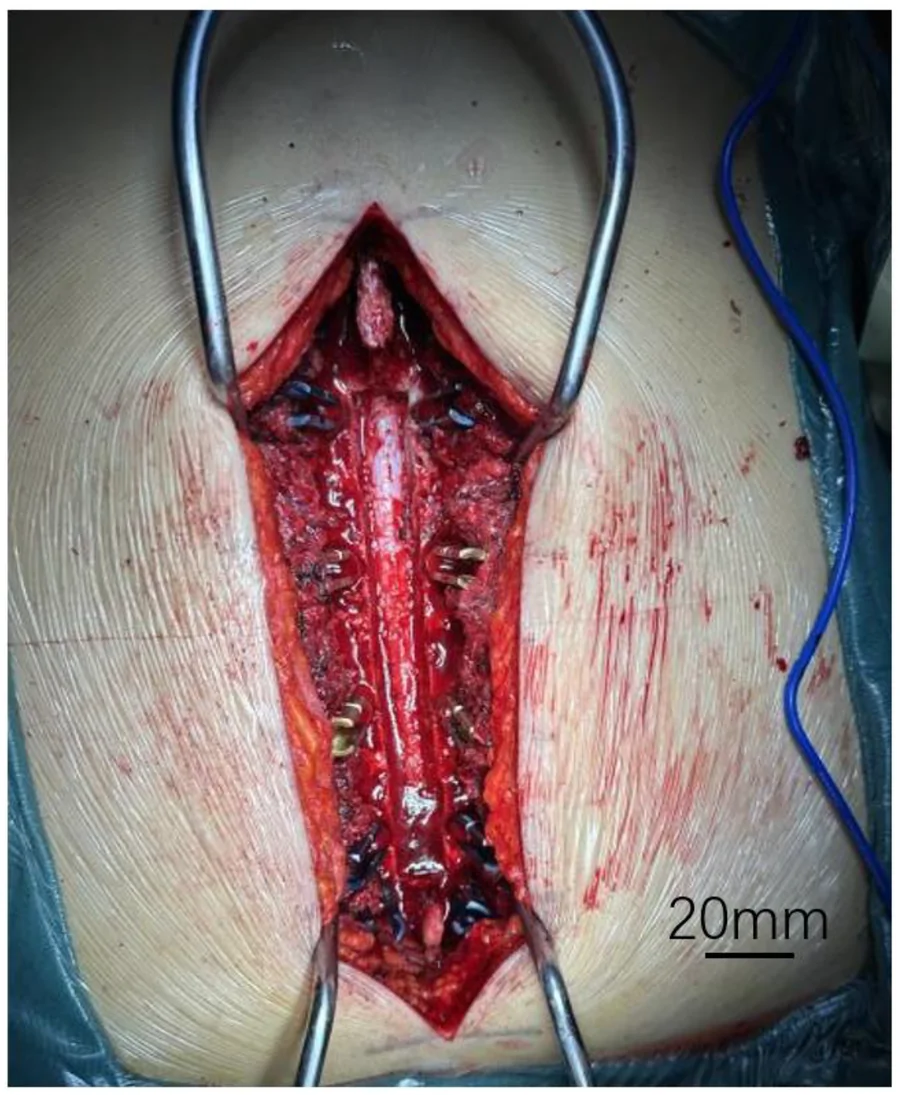
Rebounding dura after laminectomy and circumferential decompression (scale bar = 20 mm).

**Figure 4:**
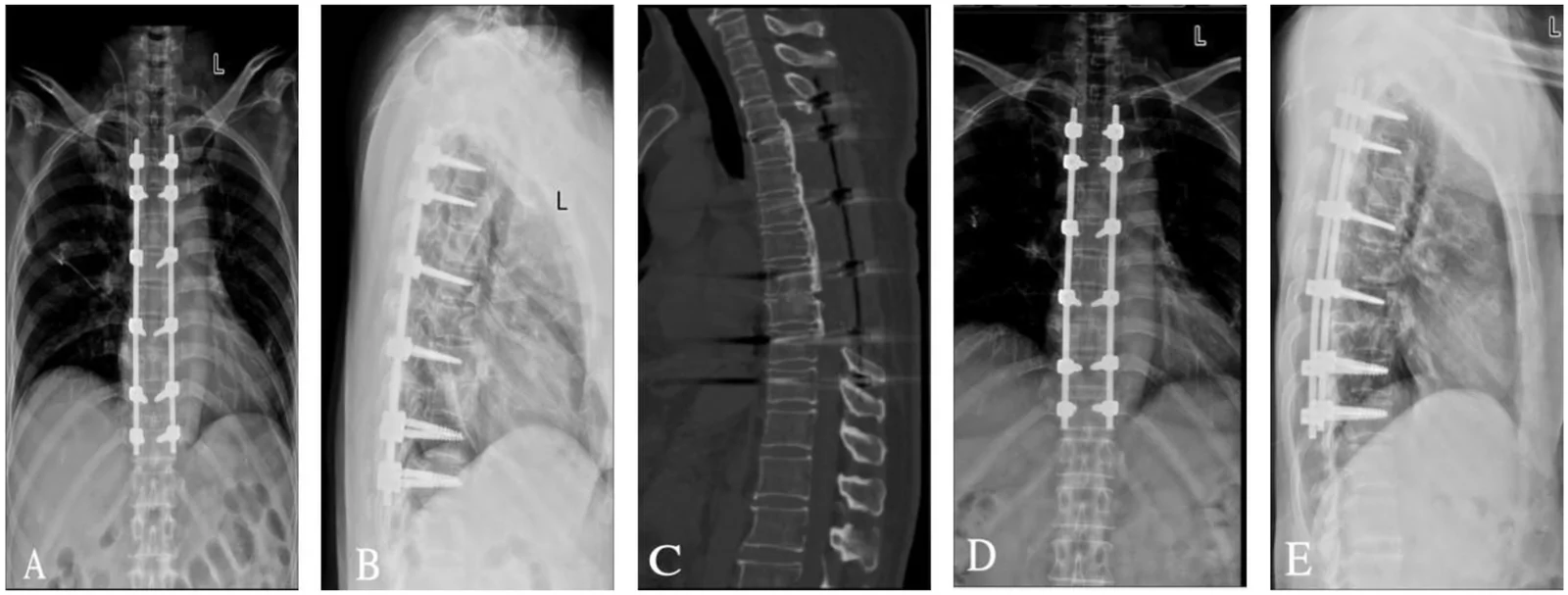
Postoperative anteroposterior **(A)** and lateral **(B)** thoracic x-rays at 1 week show good circumferential decompression range and internal fixation position. **(C)** Sagittal CT at 12-month follow-up shows good fusion at the circumferential decompression and bone graft site. Anteroposterior **(D)** and lateral **(E)** thoracic x-rays at 12 months.

Immediately following surgery, the patient reported resolution of the abdominal and perineal sensory disturbances, with additional improvement noted in bilateral lower limb sensation over the subsequent weeks. The preoperative sensory deficit was primarily localized to the right perineal region and the distal aspects of both lower limbs, corresponding roughly to the S2–S4 and L4–S1 dermatomal distributions. By the 12-month follow-up, light touch and pinprick sensation had normalized in both lower extremities, with only mild residual subjective paresthesia in the right perineal region. Neurological function was serially assessed using the mJOA scale. The preoperative score of 5 improved to 8 at 1 month, 8 at 3 months, 9 at 6 months, and 10 at the final 12-month follow-up, corresponding to a Hirabayashi recovery rate of 83.3% (Table 2). The unchanged score at 1 and 3 months indicated a temporary plateau phase in the early recovery, a pattern occasionally observed in patients with chronic thoracic myelopathy, before further improvement occurred between 3 and 6 months. At 12 months, CT and x-ray imaging confirmed solid fusion at the decompressed levels and maintained internal fixation integrity, with no evidence of neurological deterioration (Figures 4C–E).

**Table 2:** Serial neurological outcomes based on the modified Japanese Orthopaedic Association (mJOA) scale.

Time point	Total mJOA score	Lower extremity motor function	Sensory function	Bladder function	Hirabayashi recovery rate (HRR, %)
Preoperative	5	2	2	1	-
1 month postop	8	3	4	1	50.0%
3 months postop	8	3	4	1	50.0%
6 months postop	9	4	4	1	66.7%
12 months postop	10	4	5	1	83.3%

Neurological status and surgical outcomes were assessed using the modified Japanese Orthopaedic Association (mJOA). Subscores: lower extremity motor function (0–4 points), sensory function (0–6 points), and bladder function (0–1 point). Total score ranges from 0 to 11 points, with lower scores indicating more severe impairment. The Hirabayashi Recovery Rate was calculated as follows: [(postoperative score − preoperative score)/(11 − preoperative score)] × 100%. Recovery is graded as excellent (≥75%), good (50%–74%), fair (25%–49%), and poor (<25%). The identical recovery rates at 1 and 3 months reflect a temporary plateau in early postoperative neurological recovery, which is not uncommon in chronic thoracic myelopathy.

## Discussion

The surgical management of long-segment T-OPLL remains one of the most challenging endeavors in spinal surgery, with no unified protocol established to date. For patients with symptomatic multilevel involvement, surgical intervention represents the only definitive treatment option, yet the choice of surgical approach remains a primary strategic issue determining both efficacy and safety (5). Current mainstream approaches include anterior (transthoracic), posterior (laminectomy decompression with internal fixation), circumferential decompression, and combined anterior-posterior approaches, each carrying distinct indications and limitations (6).

The anterior transthoracic approach provides direct ventral decompression and is well suited for localized OPLL, but its application to long-segment lesions is limited by substantial surgical trauma, cardiopulmonary demands, and technical difficulty in managing multilevel adhesions within a deep surgical field (7, 8). The posterior approach offers a more controllable alternative for long-segment T-OPLL, avoiding thoracotomy-related morbidity while allowing safe exposure of multiple segments. Its core advantage lies in achieving both indirect decompression through laminectomy and selective circumferential decompression at critical segments when necessary, combined with long-segment instrumentation for immediate stability (9).

Multiple studies have established circumferential decompression as a key technique for achieving thorough spinal cord release in complex cases (10–12). In thoracic OPLL surgery, posterior circumferential decompression is primarily indicated for cases with severe ventral compression, CT evidence of “beak-type” or massive focal ossification, and situations where anterior surgery is contraindicated (13). A study by Liu et al. with over 5 years of follow-up on circumferential decompression for multilevel thoracic OPLL demonstrated that while the procedure's efficacy lies primarily in thorough relief of existing compression rather than halting the biological progression of ossification itself, it remains a safe and effective surgical option for symptomatic thoracic OPLL (1). For the continuous long-segment OPLL presented in this case, performing limited circumferential decompression at key responsible segments represents a reasonable strategy to achieve adequate decompression while minimizing surgical invasiveness. Simultaneously, long-segment internal fixation provides immediate spinal stability and effectively prevents postoperative kyphosis.

The surgical philosophy for complex thoracic myelopathy has shifted from simple physical decompression toward a biomechanical concept of spinal cord de-tension. This strategy combines direct ventral release with indirect longitudinal shortening. In our case, the preoperative Cobb angle was ≤25°, so we did not perform a wedge osteotomy for kyphosis correction. Instead, after selective circumferential decompression at T8–9, we carried out a parallel interbody osteotomy and filled the gap with autologous bone graft without a cage. We then applied deliberate compression across the pedicle screw-rod construct to achieve local spinal shortening. This manoeuvre reduces the longitudinal tethering force on the cord, complements the ventral decompression, and minimises direct manipulation of the already compromised neural tissue. The combined effect provides adequate neural relaxation while preserving the physiological thoracic kyphosis.

Two features of the present case warrant specific emphasis. First, the involvement of seven consecutive segments (T3–T10) is exceptionally long. Most published series on circumferential decompression for thoracic OPLL involve fewer than five segments, and the surgical complexity increases substantially with each additional level due to extended exposure, greater number of ventral adhesions, and heightened risk of spinal cord ischemia from prolonged manipulation. This consideration guided our decision to perform targeted circumferential decompression at only the most compressed level (T8–9) rather than attempting direct ventral release across all seven segments—a selective strategy that achieved adequate neural decompression while minimizing additive risks. Second, the spinal shortening manoeuvre in this case differed from wedge osteotomy, which is typically reserved for kyphosis correction when the Cobb angle exceeds 25°. Because our patient's preoperative thoracic kyphosis was well within the physiological range (≤25°), we performed a parallel interbody osteotomy at T8–9 without cage placement, followed by deliberate compression across the pedicle screw-rod construct. This achieved local spinal shortening of approximately 2–3 mm, reducing the longitudinal tethering force on the spinal cord—a core principle of the de-tension strategy—while preserving the physiological thoracic kyphosis. Recent biomechanical analyses support that posterior decompression with fusion using an appropriate fixation length prevents increased spinal cord stress during dynamic loading, lending further rationale to combining shortening with long-segment instrumentation (13, 14).

The present case involved an ultra-long segment of OPLL continuously affecting seven thoracic vertebrae from T4 to T10. Had an anterior approach been elected, it would have necessitated extensive thoracotomy, incurred substantial surgical trauma, and presented significant challenges in safely managing ventral dense adhesions across multiple segments. The posterior approach, conversely, proved ideally suited to the treatment requirements of this case for several reasons. First, it allowed exposure and initial decompression (laminectomy) of all seven compressive segments through a single posterior midline incision. Second, following initial spinal cord decompression, precise circumferential decompression could be performed under direct vision using an ultrasonic bone scalpel at the most severely compressed T8–9 segment. This involved accurately grinding along the ossification margins, patiently separating adhesions with a nerve dissector, releasing the critical point of compression, and performing bone graft fusion to maintain spinal canal volume and prevent re-compression. Third, long-segment fixation from T3 to T11 provided solid immediate stability for the spine after multilevel decompression. Thus, this surgical approach achieved an optimal balance among “thorough decompression,” “surgical safety,” and “spinal stability,” representing a rational and necessary choice for this complex pathology.

In the present case, a small dural tear occurred during separation due to severe adhesion of OPLL at T8–9. Intraoperative sealing was achieved with gelatin sponge compression, and following 5 days of strict bed rest and controlled drainage management, the leak healed successfully without sequelae. Iatrogenic dural tear is a well-recognized complication of posterior thoracic spine surgery, with revision surgery, severe epidural adhesions, and OPLL with dural ossification identified as major risk factors (15). Espiritu et al. observed that once a dural tear occurs, immediate intraoperative management is required; small tears can be directly sutured, while large or irregular defects often require sealing with fibrin glue combined with gelatin sponge, with refractory cases potentially necessitating lumbar drainage (16). The successful outcome in this case confirms that for appropriately managed minor dural tears, systematic conservative management can effectively obviate the need for revision surgery, underscoring the importance of both risk anticipation and structured management protocols in complex spine surgery.

Given the inherent complexity of long-segment thoracic OPLL in clinical practice, this case aims to deepen understanding of the treatment challenges associated with this disease by providing detailed documentation of surgical decision-making and technical nuances. Existing literature indicates that for such complex OPLL, the timing of surgery and choice of surgical approach remain subjects of ongoing controversy. Some patients experience delayed surgical treatment due to atypical early symptoms or underestimation of disease progression speed; such delays in diagnosis and treatment may increase surgical difficulty and adversely affect final neurological recovery. Although some studies suggest that conservative treatment may be attempted for select mild or stable cases, for patients presenting with progressive myelopathy as in this instance, timely surgical intervention remains the key to preventing irreversible neurological damage (17–19). The preponderance of evidence indicates that with individualized, adequate decompression and stabilization surgery, patients can achieve favorable neurological improvement at medium- to long-term follow-up (14, 20). In the present case, posterior circumferential decompression and long-segment fixation resulted in significant neurological recovery, further supporting the position that active and precise surgical treatment is both safe and effective for symptomatic long-segment thoracic OPLL.

## Conclusion

Thoracic ossification of the posterior longitudinal ligament (T-OPLL) represents a progressive pathological entity that predominantly affects middle-aged and elderly populations, typically manifesting as gradually worsening bilateral lower extremity numbness, weakness, and in advanced stages, incomplete paralysis. The diagnosis relies on characteristic clinical signs of myelopathy corroborated by CT and MRI evidence demonstrating abnormal ossification of the posterior longitudinal ligament with secondary severe spinal canal stenosis and spinal cord compression. In accordance with existing clinical guidelines and the preponderance of published literature, for patients presenting with unequivocal neurological deficits, surgical decompression constitutes the fundamental therapeutic intervention to prevent irreversible neurological damage (1, 18). The present case report, documenting a complex instance of ultra-long segment OPLL continuously involving seven vertebral levels successfully managed with posterior circumferential decompression combined with long-segment internal fixation, provides further confirmation that for symptomatic long-segment thoracic OPLL, timely and thorough surgical intervention guided by precise imaging assessment represents a key and effective strategy for achieving neurological recovery and preventing long-term disability.
